# Insights Into *SND1* Oncogene Promoter Regulation

**DOI:** 10.3389/fonc.2018.00606

**Published:** 2018-12-11

**Authors:** Begoña Ochoa, Yolanda Chico, María José Martínez

**Affiliations:** Department of Physiology, Faculty of Medicine and Nursing, University of the Basque Country UPV/EHU, Leioa, Spain

**Keywords:** staphylococcal nuclease and Tudor domain containing 1, Tudor-SN, transcriptional regulation, chromosome 7 band 7q.32.1, miR-593, SND1-IT1, oncogene promoter

## Abstract

The staphylococcal nuclease and Tudor domain containing 1 gene (*SND1)*, also known as *Tudor-SN, TSN* or *p100*, encodes an evolutionarily conserved protein with invariant domain composition. SND1 contains four repeated staphylococcal nuclease domains and a single Tudor domain, which confer it endonuclease activity and extraordinary capacity for interacting with nucleic acids, individual proteins and protein complexes. Originally described as a transcriptional coactivator, SND1 plays fundamental roles in the regulation of gene expression, including RNA splicing, interference, stability, and editing, as well as in the regulation of protein and lipid homeostasis. Recently, SND1 has gained attention as a potential disease biomarker due to its positive correlation with cancer progression and metastatic spread. Such functional diversity of SND1 marks this gene as interesting for further analysis in relation with the multiple levels of regulation of SND1 protein production. In this review, we summarize the *SND1* genomic region and promoter architecture, the set of transcription factors that can bind the proximal promoter, and the evidence supporting transactivation of *SND1* promoter by a number of signal transduction pathways operating in different cell types and conditions. Unraveling the mechanisms responsible for *SND1* promoter regulation is of utmost interest to decipher the SND1 contribution in the realm of both normal and abnormal physiology.

## Introduction

**Figure 1:**
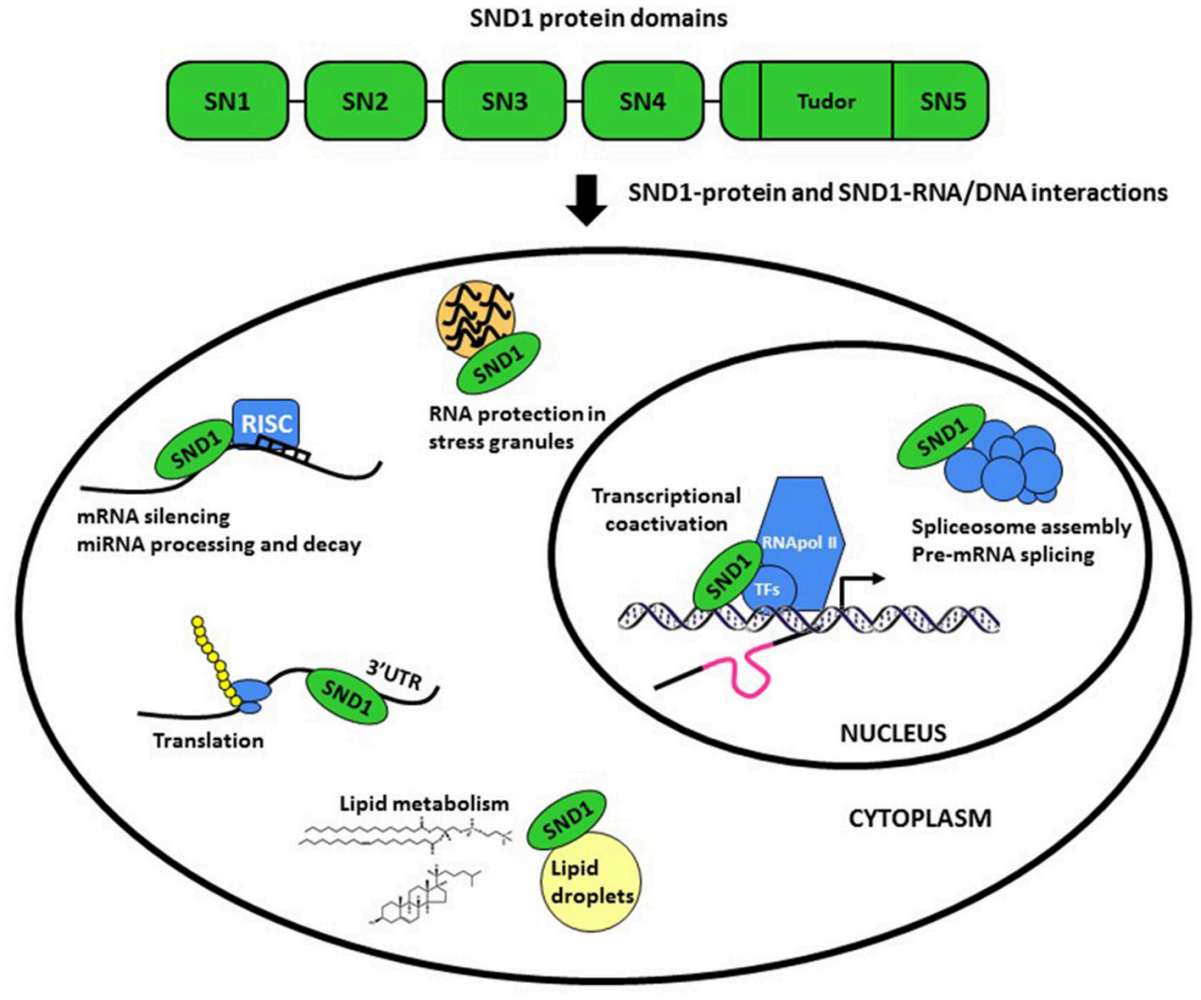
Schematic representation of the structure and functions of SND1 protein. The protein comprises a tandem of four Staphylococcal nuclease-like domains (SN1-4) followed by a fusion of an entire Tudor domain and a truncated SN domain (SN5). SN and Tudor domains enable SND1 for interacting with RNA/DNA and proteins to operate in a plethora of processes.

Staphylococcal nuclease (SN) and Tudor domain containing 1 (*SND1*) gene encodes SND1, an evolutionarily conserved multidomain protein also called Tudor-SN, TSN or p100 (1–4). The invariant domain composition of SND1 comprises a tandem of four SN-like domains at the N-terminus (SN1-4) followed by a C-terminal domain formed by a fusion of a Tudor domain and a truncated SN domain (5) (Figure 1). The potent interaction capacity of SN domains with RNA (6) and of the Tudor domain with modified (methylated) aminoacids (7, 8), together with its intrinsic endonuclease activity (9) for degrading double-stranded RNA (10) enable SND1 for participating in an unusual multiplicity of functions (summarized in Figure 1). We refer the reader to some recent reviews addressing the biochemistry and function of this protein (11–13).

It has now become clear that the SND family members behave as global regulators of gene expression. SND1 was first characterized as a transcriptional coactivator, interacting with several transcription factors, including EBNA2 (5, 14), c-Myb (15), STAT6 (16), STAT5 (17), E2F1 (18), and PPARγ (19). Moreover, the SND1-mediated post-transcriptional regulation is surprisingly diverse. It includes spliceosome assembly and pre-mRNA splicing (20, 21), RNA interference, stability and editing (6, 9, 10), microRNA (miRNA) decay (22), and RNA protection in stress granules (23, 24). In addition, it has been illustrated a role for SND1 in the regulation of protein synthesis, ubiquitination and proteasomal degradation (25, 26).

Clinical and experimental studies show a close association between overexpression of SND1 and progression and aggressiveness of a spectrum of common cancers that include colon, breast, prostate, lung, glioma, melanoma, and liver cancer (27–34). Using different models, researchers have identified a host of mechanisms through which SND1 modulates prosurvival and proliferative genes promoting carcinogenesis. Of particular relevance are the networks involving NF-κB activation and miR-221 induction (32), miR-184 expression and JAK/STAT3 inhibition (34), TGFβ1/Smad signaling pathway (25), Wnt/β-catenin activation (27), ERK, and Akt activation (35, 36), as well as the interaction of SND1 with partner proteins like metadherin-1 (30, 37) and monoglyceride lipase (26).

Since full or organ-selective SND1-knockout animal models are not yet available, the systemic role of SND1 remains to be fully delineated. Several works have shown that knocking down endogenous SND1 inhibits proliferation in diverse cancer cell lines (23, 29, 37–39). As physical interaction of SND1 with E2F1 is critical for the G1/S phase transition (18), the cell cycle arrest in SND1-knockout cells has been attributed to the absence of the SND1-driven endonucleolytic degradation of specific miRNAs that downregulate crucial proteins for the G1/S transition, including E2F1 (18, 40). In addition, other studies have reported that SND1 knockdown fosters apoptosis (41), reduces RISC activity (29, 37), and impairs the aggregation of stress granules (23).

The oncogenic functions assigned to SND1 might be also regarded as a result of its ubiquitous expression in tissues (42) and its ample capacity for interacting with nucleic acids, individual proteins, and protein complexes in the cytoplasmic (43–45) and nuclear compartments (46). Consistent with this, we recently documented that, in human hepatoma HepG2 cells, nuclear SND1 interacted with the genomic DNA to hook SND1 on the promoter of a broad number of target genes modulating cell growth, oncogenic transformation, viral infection and metabolic regulation (47).

Extensive rewiring of lipid metabolism and lipogenic phenotypes are features that distinguish cancer cells from normal cells (48, 49). Besides providing energy and building units for the newly forming cells, lipids generate a network of protumorigenic signals that promote tumor growth (49). Strong evidence support a role for SND family members in lipid homeostasis. Particularly illustrative are their implication on specific aspects of lipid bodies biogenesis and the secretion of milk lipids by mammary epithelial cells (43, 50), the secretion of lipoprotein particles by liver parenchymal cells (45, 51), and the expression of genes regulating glycerophospholipid homeostasis and phosphatidylcholine content in HepG2 cells during inflammation (47). Interestingly, recent findings have revealed that SND1 overexpression alters cholesterogenesis and limits triglyceride synthesis via modulation of the regulatory cholesterol pool in endoplasmic reticulum, thus suggesting that SND1 may be decisive to determine events that modify the permeability properties of cancer cell membranes and facilitate cell proliferation (52, 53).

Despite these and other major advances, the precise function of SND1 in normal and challenged cells is not fully clarified. SND1 function and *SND1* transcription regulation are inseparable. Nevertheless, as is often the case, there is a surprisingly limited knowledge of the molecular mechanisms and agents that regulate SND1 production at the transcription level under the above-mentioned pathophysiological conditions.

After briefly summarizing the *SND1* genomic context, the present review will mainly focus on the human *SND1* promoter characterization. We provide a comprehensive description of the transcription factors and signal transduction pathways that trigger *SND1* promoter activation in different cell types and conditions.

## The Human *SND1* Gene

Orthologues of *SND1* are found in mammals (5, 14, 54), plants (4), *Drosophila, C. elegans* (9), and many other species. The main characteristics of the gene, transcript, protein and gene promoter for *Homo sapiens* and for the two most common rodents *Mus musculus* and *Rattus norvegicus* are shown in Table 1. The genomic context of human *SND1* is the large arm of the chromosome 7 (7q32.1), while *Snd1* is assigned to chromosome 4 (4q23) in the rat (55) and to chromosome 6 in the mouse. It is worth mentioning that the large arm of human chromosome 7 is associated with chromosomal instability and many types of neoplasia (56).

**Table 1:** General information of *SND1* gene, transcript, protein and promoter in humans, mouse and rat.

	***Homo sapiens*** **(human)**	***Mus musculus*** **(house mouse)**	***Rattus norvegicus*** **(Norway rat)**
Gene name	SND1	Snd1	Snd1
Gene ID	ID: 27044	ID: 56463	ID: 64635
Aliases	TDRD11, Tudor-SN, p100	AL033314, Tudor-SN	SND p102
Chromosome	7	6	4
Location	NC_000007.14 (127651989.128092609)	NC_000072.6 (28480315.28935162)	NC_005103.4 (55772377.56171669)
Length (bases)	440,621	454,848	399,293
% Identity vs. human		87	84
Exon counts	24	24	24
mRNA ID	NM_014390.3	NM_019776.2	NM_022694.2
Length (bases)	3,678	3,482	3,475
% Identity vs. human		87	87
Protein ID	NP_055205.2	NP_062750.2	NP_073185.2
Aminoacids (number)	910	910	909
% Identity vs. human		97	97
Promoter	EF690304	-	AY957585
Length (bases)	3,808	4,600	1,688
% Identity vs. human, Region (−300, +20)		82	84
CpG island	904 bp (−555, +349)	850 bp (−532, +318)	912 bp (−394, +518)
G-quadruplex (number), Region (−300, +20)	4	2	3

*Data are extracted from https://www.ncbi.nlm.nih.gov/ database and analyzed using BLAST, UCSC Genome Browser (Dec 2013 GRCh38/hg38) and GQRS Mapper tools*.

The human and rodent genes expand 400-455 kb and contain 24 exons with identical organization, and conserved intron-exon boundary sequences in agreement with the canonical GT/AG rule. The transcription initiation site of the human gene was mapped at exon 1, while the rat *Snd1* gene pointed to the existence of two different transcription start sites, the main one being assigned to the G at position 216 upstream of the ATG codon and the secondary start point 34 bp downstream (57). *SND1* encodes 16 splicing variant transcripts, one of them translates into the full SND1 protein of 910 amino acids, other codes for a 231 amino acid protein shortened in the two extremes -uncharacterized- and the others are noncoding transcripts. The mouse and rat Snd1 proteins contain 910/909 amino acids, respectively. Sequences are 90% identical between human, rat and mouse genes and reach 97.4% for the proteins.

**Figure 2:**
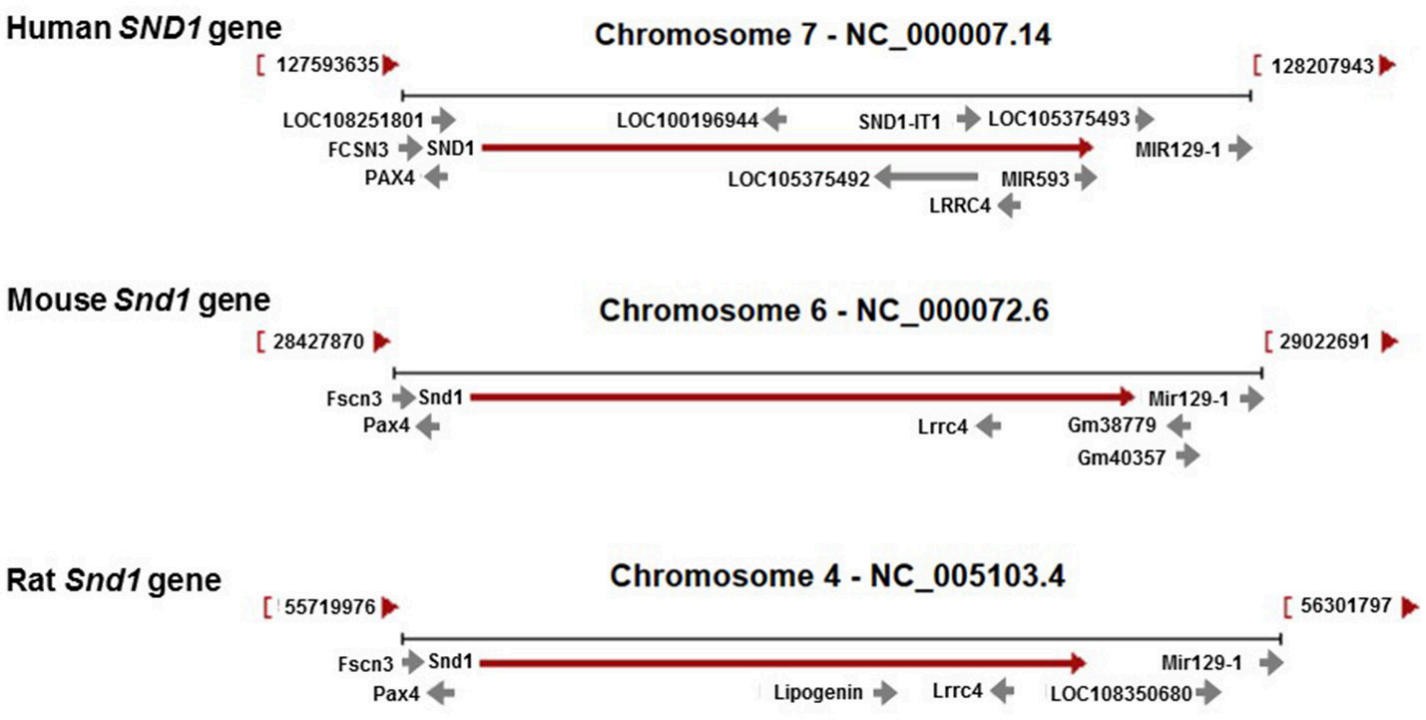
Genomic regions for the human *SND1* and the rat and mouse orthologues. Contexts are well conserved in the three species regarding *PAX4, FCSN3* and *Mir129-1*. The *SND1* gene hosts *LRRC4* while the intronic genes *MIR593* and *SND1-IT1 (*SND1-intronic transcript 1) are present only in the human genome in sense orientation.

The genomic landscapes of the three orthologues are partially similar (Figure 2). Upstream, antisense *PAX4* and sense *FSCN3* and, downstream, sense *miR-129-1* are conserved. Complexity of intragenic elements is far greater in the human compared with the murine genes. Although *LRRC4*, a gene involved in the normal development and tumorigenesis of the nervous system, is shared by the three orthologues in antisense orientation, there are two overlapping intronic elements in the same strand orientation as the host gene that are unique to human *SND1*: *miRNA-593* and the *SND1* intronic transcript 1 (*SND1-IT1*). These elements map at introns 17 and 16, respectively.

Despite being defined as a long noncoding RNA gene, *SND1-IT1* has the capacity of encoding an uncharacterized protein called brain and nasopharyngeal carcinoma susceptibility protein NAG8. Low levels of a different noncoding intronic transcript derived from *SND1* have been detected in clear cell renal carcinoma (58). This novel transcript maps at the yet uncharacterized intron 14.

Using the genomic resources miRBase and Ensembl, Godnic et al. identified 27 miRNA/host gene pairs with cross-species conserved location (59). Among them, the pair *miRNA*-*593*/*SND1* is of particular interest for being a miRNA gene located within a gene encoding for a component of the miRNA silencing machinery. The *miR-593* gene has been linked to certain diseases, including breast cancer (60), esophageal cancer (61), and pediatric medulloblastoma (62), and is considered a non-invasive diagnostic biomarker for children with combined pituitary deficiency (63). Likewise, Fan et al. demonstrated a role for the BRCA1-mir-593-5p-MFF axis in cisplatin sensitivity in tongue squamous cell carcinoma (64).

Whether transcription initiation regions of intragenic miRNAs are into or separate from the promoter of their host genes is a matter under intense study. Approximately 50% of the intragenic miRNAs are known to be expressed from the introns of their host genes (65), whereas about one-third of the intronic miRNAs have transcription initiation sites different from the host gene promoter (66). Intriguingly, the miRNA-593-5p is an intronic miRNA in the *SND1* gene whose BRCA1 binding site is within the *SND1* gene sequence (64). In analyzing the 5-kb region upstream of *miR-593-5p*, Fan et al. found that BRCA1 transactivated indeed miR-593-5p expression increasing its promoter activity. By contrast, they observed that BRCA1 overexpression failed to alter substantially SND1 mRNA levels (64), suggesting that this miRNA/host gene pair does not share the transcription start sites.

## The *SND1* Promoter

Understanding a protein-coding gene promoter not only provides a deep insight into the regulation of gene expression but also sheds light on the protein function. We will review the current knowledge available on the *SND1* promoter architecture and the set of transcription factors that bind the proximal promoter. We also describe an *in silico* analysis of the quadruplex forming G-rich sequences that could alter *SND1* promoter shape and activity.

### Basal *SND1* Expression

Our own previous studies provided the isolation and characterization of a 3,808 bp sequence of the human *SND1* gene promoter (GenBank ID: EF690304) (67), and a region of 1,688 bp corresponding to the rat *Snd1* promoter (GenBank ID: AY957585) (57). Comparing the *Homo sapiens* and *Rattus Norvegicus* genome databases we found 84% identity in a 300 bp region upstream to the transcription start point of the isolated promoter sequences of *SND1* (Table 1). Similar identity (82%) was found in the same region of the 5′ flanking region of the mouse *Snd1* (Table 1). The high sequence similarities between species point to the presence of conserved pattern of *cis*-elements that are critical for the protein expression regulation along evolution.

Bioinformatic analysis of the promoter sequences revealed multiple *cis*-acting elements and potential binding motifs for transcription factors relevant in regulating *SND1* transcription under basal and activated cellular states. We identified features characteristic of housekeeping genes, such as the absence of the canonical TATA box and the presence of CCAAT boxes and a large number of GC-sites (57, 67, 68). Most TATA-less promoters are enriched in CpG islands and CCAAT boxes near the transcription start site in specific locations for recruiting and stabilizing the transcriptional machinery. Human and rat gene promoters possess one CpG island in the region (−555, +349) and (−394, +518) respectively, with multiple GC boxes for the functional binding of Sp1 transcription factor (67, 68). Both promoters also contain two reverse CCAAT boxes, common elements in the eukaryotic genes that usually appear in the (−80, −40) region of promoters with or without TATA box (69). We demonstrated that these motifs are binding sites for NF-Y by using immunoprecipitated chromatin from human and rat hepatoma cells (57, 67, 68). Therefore, every one of the CCAAT boxes and GC boxes are regulatory elements likely required to maintain the basal expression of SND1 in most tissues.

**Figure 3:**
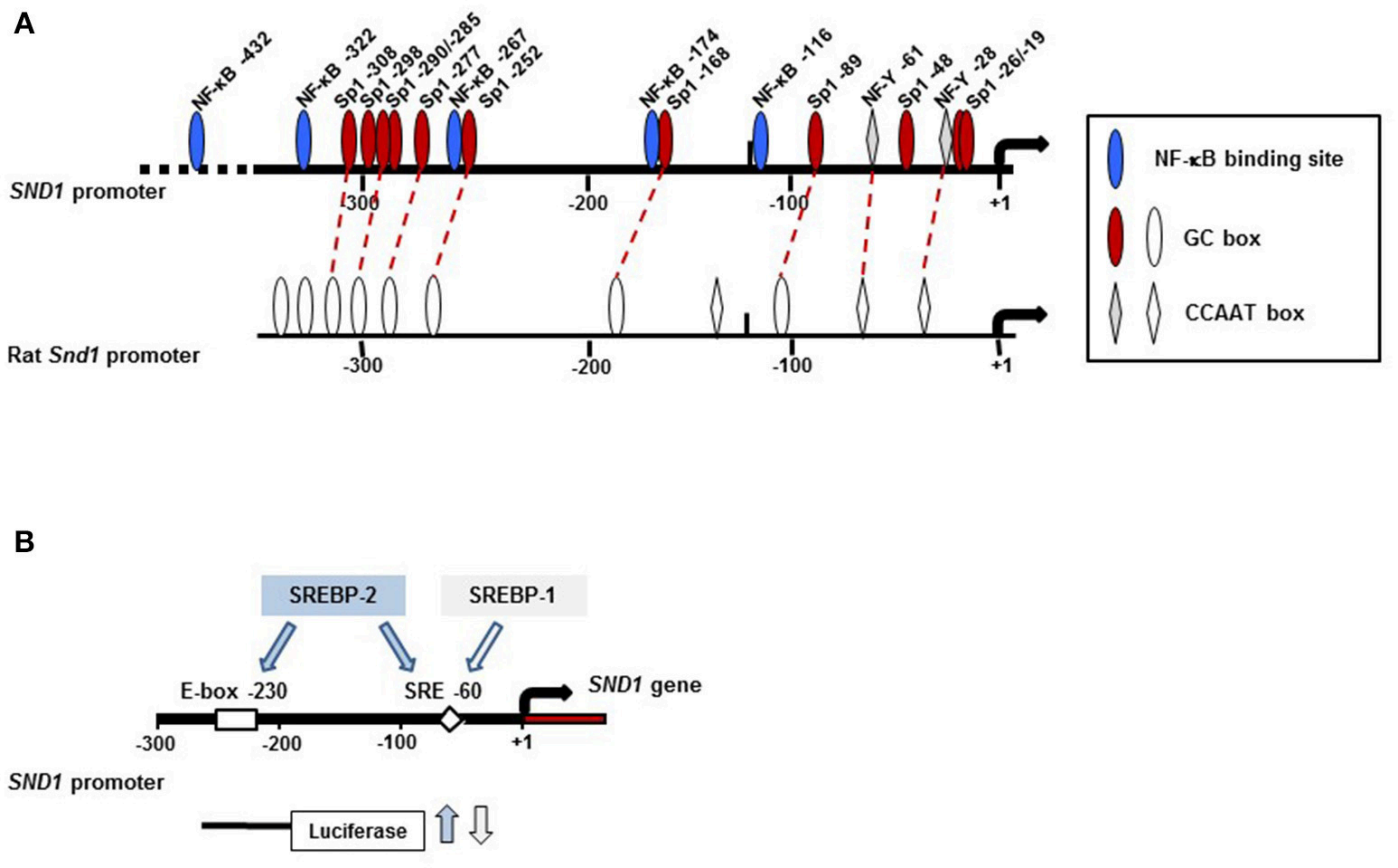
Location of key regulatory elements of the human *SND1* proximal promoter. **(A)** Diagram of region (-400, +10) of the promoter. Promoter activity is under the control of the transcription factors Sp1, NF-κB and NF-Y with 11 (GC boxes, red and white ovals), 5 (NF-κB elements, blue ovals) and 2 (CCAAT boxes, gray and white diamonds) binding sites identified, respectively. The rat promoter conserves some of these regulatory sites. **(B)** Promoter activity is increased by SREBP-2 binding to E-box −230 and/or SRE −60 whereas it is inhibited by SREBP-1 binding to SRE −60. The arrow indicates the transcription initiation site.

Promoter activity of 5′-deletion fragments determined by luciferase reporter assays in human and rat hepatoma cells revealed a minimal promoter at −112 bp and an enhancer region between −112 and −274 bp that accounted for the maximal transcriptional activity of the proximal promoters (67, 68). As Figure 3A shows, this region contains two CCAAT boxes at position −28 and −61 in the human promoter and six GC boxes conserved in the rat homologue that are recognized by NF-Y and Sp1 and assess the efficient transcription of the *SND1* gene family. Functionality of these elements was confirmed in mutation experiments (67, 68).

### Regulatory Elements Associated With *SND1* Expression

Beyond the conserved *cis*-elements in *SND1* proximal promoter, we characterized a number of regulatory elements that control *SND1* promoter activity in adaptation to cell environment changes. These elements receive inputs from a multitude of stimuli and signaling pathways through a customized collection of transcription factors that might affect *SND1* expression. Emerging information from plants (4) and animal cells has demonstrated that SND1 protein expression changes in response to cellular stresses related to inflammation, tumor growth, hypoxia, heat shock, oxidative conditions, and DNA damage (12, 70, 71).

Cellular stress results in an inflammatory response mainly triggered by inflammatory cytokines such as TNFα and mediated by the activation of NF-κB cascade. NF-κB is the key transcription factor that induces the expression of protective genes and finally resolves the protection or death of the cell (72, 73). We reported the activation of *SND1* transcription and the nucleocytoplasmic redistribution of SND1 protein during the inflammatory response prompted by TNFα in human hepatoma cells (47, 67). Upregulation of SND1 involved direct binding of NF-κB to five target sites in the proximal promoter, as shown in Figure 3A (67). This response seems to be human-specific, as, although the NF-κB binding site motif is conserved in the rat promoter, NF-κB did not interact with that region. Whether SND1 displays a protective role for liver cells under inflammatory conditions is an intriguing possibility that warrants future in-depth studies.

Inflammation is linked to endoplasmic reticulum (ER) stress through a crosstalk between NF-κB, ATF6 and XBP1 signaling pathways (74). In response to ER stress, eukaryotic cells trigger various signaling cascades termed the unfolded protein response (UPR) for controlling gene transcription and switching off translation initiation. Non-translating cellular mRNAs are sequestered into cytoplasmic stress granules waiting for a later translation or degradation (75). In this sense, it is worth mentioning that SND1 has been defined as an important protein in RNA protection due to its ability to interact with protein components of the cytoplasmic stress granules (12, 23, 24, 70, 71, 76). We presented experimental data of SND1 protein expression and *SND1* promoter activity upregulation by pharmacological ER stress in HepG2 cells (77). We encountered a number of potential ER stress response elements in *SND1* promoter and by plasmid-mediated ATF6 overexpression demonstrated the participation of the transcription factor ATF6 in the transcriptional activation of *SND1* gene (77). ATF6 is a membrane-bound transcription factor that plays a central role in the UPR. When agents disrupt the folding of proteins in the ER, ATF6 is cleaved by sequential proteolysis to release its cytoplasmic domain, which enters the nucleus and activates protective gene transcription. Therefore, *SND1* can be added to the selective list of ATF6 target genes.

Given that the cellular processing of ATF6 activation is analogous to that undergone by sterol regulatory element binding proteins (SREBPs) in response to cholesterol deprivation, parallel activation of ATF6 and SREBP-2 in animal cells (78) may be envisioned.

In this regard, our own work revealed that *SND1* is a target gene for SREBPs. The activation of SREBP-2 in HepG2 cells cultured in the presence of the cholesterol synthesis inhibitor simvastatin or in a lipoprotein-deficient medium resulted in an increment of *SND1* promoter activity and transcript levels (79). ChIP and mutational assays confirmed the functional binding of SREBP-2 to two sites in the proximal promoter sequence containing the SRE −60 and E-box −230 motifs while SREBP-1 binding is restricted to the region with the regulatory element SRE −60 (Figure 3B). SREBP-gain-of function and loss-of function experiments confirmed the binding of endogenous SREBP-2 and SREBP-1 to specific sites on the *SND1* promoter. To our surprise, binding regulated *SND1* transcription in opposite ways: induction by SREBP-2 and repression by SREBP-1 overexpression (79). Such contrasting effects suggest that SND1 expression responds to particular upstream signals recruiting selectively SREBP-1 and/or SREBP-2 in concert with a set of transcription factors to *SND1* promoter. We now know that the optimal action of SREBPs usually requires the cooperation with other transcription factors, commonly Sp1 and NF-Y (80). The promoter of *SND1* contains conserved sites for Sp1 and NF-Y quite close to SRE −60. Preliminary findings indicate that, whereas the SREBP-2-driven activation of *SND1* promoter is independent of Sp1 and/or NF-Y, the participation of NF-Y is required for the inhibition of the *SND1* promoter activity by SREBP-1 (79). Contribution of SND1 and SREBPs to lipid metabolism reprogramming in cancer cells may be, otherwise, expected in view of the report illustrating that deregulated cholesterol homeostasis and SREBP-2 activity is associated with SND1 overexpression (52). We need to keep in mind that this setting facilitated hepatic cancer cell proliferation.

**Figure 4:**
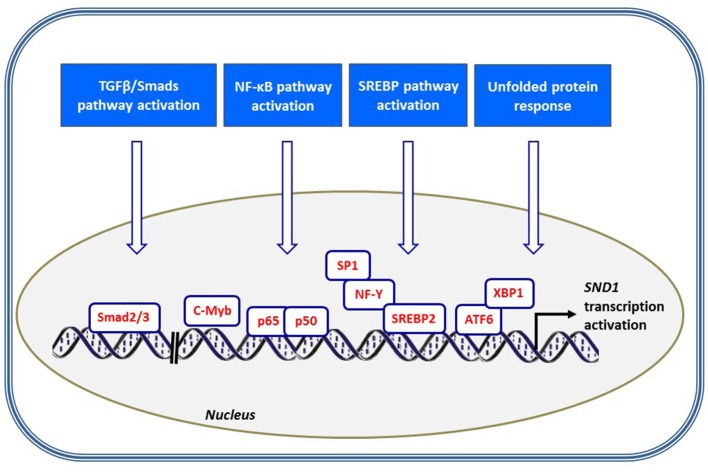
Scheme illustrating the signaling pathways and transcription factors contributing to *SND1* promoter activation. *SND1* is transcriptionally upregulated by several stress settings, including TGF/Smads pathway through Smad2/3 binding, NF-κB-mediated inflammation, SREBP activation concomitant to cholesterol depletion and ATF6/XBP1-mediated endoplasmic reticulum stress.

A recent study reported a close association between SND1 upregulation and TGFβ1/Smad signaling pathway activation in order to promote epithelial-to-mesenchymal transition in breast cancer (25). Mechanistically, TGFβ1 triggers the activation of Smads' transcription factors and the Smad2/Smad3 complex binds specific recognizing sites located in the distal promoter of *SND1* at region (−1,100, −1,000) and (−2,100, −2,000) and enhances the transcriptional activity (25) (Figure 4). The downstream action of SND1, functioning as a coactivator, finally results in the disruption of cytoskeletal organization and increased cell motility and invasion and metastasis of breast cancer.

Evidence of a direct binding of c-Myb to the promoter of human *SND1* has been provided by ChIP-on-chip assays (81). c-Myb is a key transcription factor in proliferation, differentiation, and cell survival. It is activated in certain human leukaemias and in solid cancers such as colon and breast cancer (82). In breast cancer, c-Myb interacts with transcriptional cofactors and binds to E-box and c-myb sites on the gene promoters to activate their transcription (83). By similarity, it is tempting to speculate that c-Myb may recognize certain E-box motifs within the promoter sequence of *SND1* and modulate the transcription of the gene. Nevertheless, the regulatory role of c-Myb over *SND1* promoter activity, if any, remains to be confirmed.

The ensuing conclusion is that SND1 expression is modulated by growth factors, inflammatory cytokines, ER disruptors, and metabolic stress, all signals addressed by the transcription factors Smads, NF-κB, ATF6, and SREBPs, and other factors still to enumerate (Figure 4). Altogether, these findings suggest the involvement of SND1 in diverse pathways that are dysregulated in oncogenic processes. In other words, cells handle external stimuli that condition the outputs and the program of factors and cofactors that modulate the *SND1* promoter activity and the level of expression of SND1. Setting the level of SND1 is crucial. A more comprehensive insight into regulation of *SND1* transcription will require understanding how the *cis*- and *trans*-regulatory elements work as a unit in the promoter.

### Alternative DNA Conformations Affecting *SND1* Expression

A corollary of the above is that some aspects of gene expression are under the control of transcription factors and *cis*-acting elements in the gene promoter region in a highly dynamic manner. In addition, features beyond the primary sequence of DNA, including DNA shape and flanking sequences, can modulate binding specificity of a transcription factor to distinct sites (84). For instance, DNA sequences enriched in GC are susceptible to adopting non-B-DNA conformations such as single-stranded DNA and G-quadruplex structures. An intriguing observation is the significant enrichment of DNA G-quadruplex-forming motifs in most oncogene promoters (85).

A G-quadruplex is a four-stranded DNA structure that is composed of stacked guanine tetrads linked by loop nucleotides that can differ in their folding patterns (86). The heterogeneity of G-quadruplex structures and the potential therapeutic applications as targets for antitumor drugs have attracted intense attention during the last years (87–89). Thus, identification of G-quadruplex structures in human genes is continuously growing. G-quadruplexes in promoter regions act as important modulators of transcriptional regulation and can activate or repress gene expression (89, 90). As *SND1* is considered an oncogene, we interrogated *SND1* promoter for the possibility of G-quadruplex structures. Here we report the findings for the first time.

**Figure 5:**
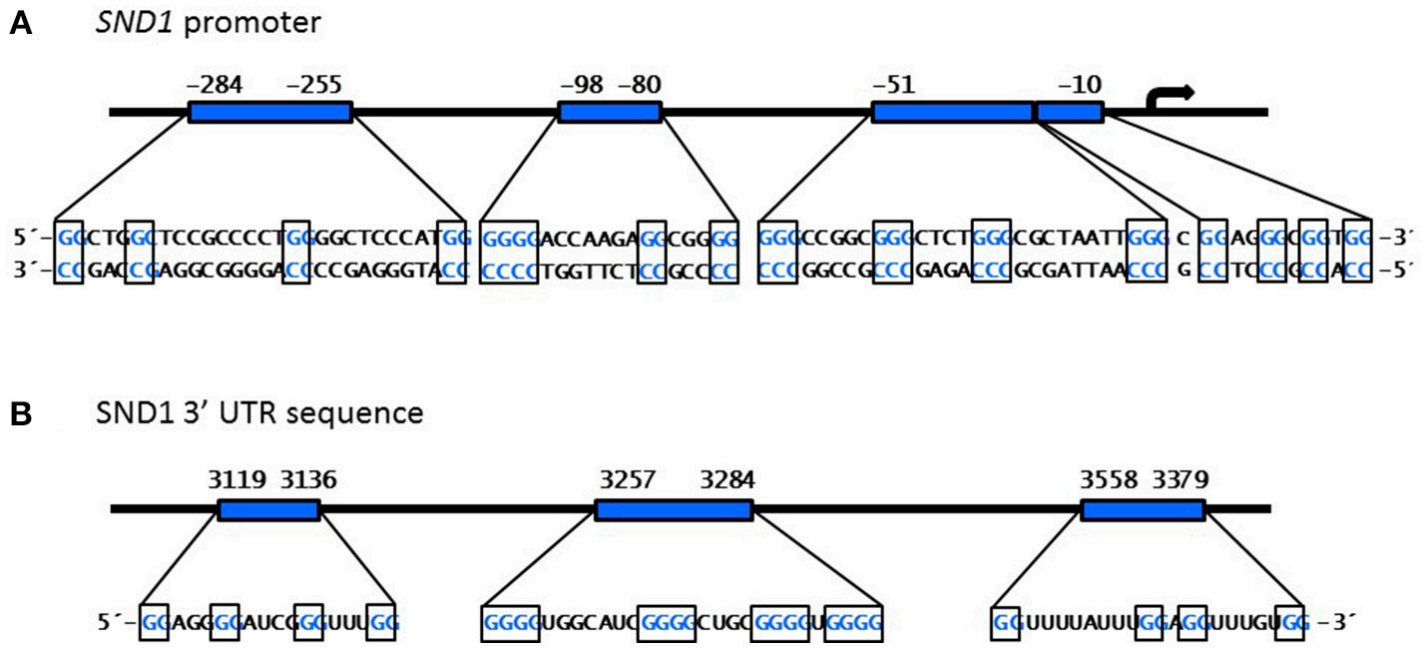
Predicted G-quadruplex structures in the *SND1* proximal promoter and the 3′UTR sequence. *In silco* prediction and distribution of the quadruplex forming G-rich sequences (each G-group in boxes) found by QGRS Mapper program (91) in the nucleotide sequence of human *SND1* promoter **(A)** and 3′ untranslated region **(B)**.

A set of *in silico* algorithms are available for predicting G-quadruplex structures in DNA or RNA nucleotide sequences. By using the web-based server QGRS Mapper (91) and QuadBase2 (92) we found the potential to form four G-quadruplex structures in the *SND1* proximal promoter (Figure 5A and Table 1). One exclusive sequence found at −51 in the proximal promoter of *SND1*, but not in the rat and mouse orthologues, was the best candidate to form a stable G-quadruplex structure according to the high computed scoring. The predicted structures occur in a DNAse I hypersensitivity peak located at (−287, +3) by the ENCODE group at UCSC Genome Browser (GRCh38/hg38). In general, nuclease hypersensitive sites provide information about the active transcriptional regulatory elements (93). It is particularly interesting that, in this GC-rich region, there are placed various conserved GC boxes recognized by Sp1 (Figure 3A). Whether G-quadruplexes formation masks some specific Sp1 binding sites or, instead, it confers a new binding site for an alternative factor onto *SND1* promoter and their consequences on SND1 expression require further in-depth studies.

Figure 5B shows that there are also several putative G-quadruplex forming motifs in the 3′ UTR sequence of the SND1 transcript. G-quadruplex structures might affect miRNA binding sites and, therefore, alter SND1 mRNA posttranscriptional processing.

Again, there is limited information relative to the posttranscriptional regulation of *SND1* gene. We know that SND1 mRNA is modulated by microRNAs. Of particular clinical interest is the identification of miR-361-5p, miR-184 and miR-320a targeting the 3′ UTR of the SND1 transcript. It has been illustrated that overexpression of either miR-361-5p, miR-184, or miR-320a decreased cancer cell viability, migration and invasion through direct downregulation of *SND1* oncogene in gastric and colorectal cancer (94), in malignant glioma and breast cancer (34, 95) and in lung cancer cells (96). These findings rise the question about the diagnostic relevance of these miRNA/SND1 axes and their therapeutic application as a tumor suppressive strategy for cancer treatment.

## Conclusions and Future Perspectives

We are currently lacking a thorough examination about the precise role of SND1 in normal and challenged cells. A detailed map of the SND1 transcriptional regulation is needed. Here we illustrate the promoter architecture of *SND1* and the set of transcription factors that modulate the promoter activity and the level of transcription of SND1. It is relevant that SND1 expression is under the control of transcription factors, such as NF-Y, Sp1, Smads, NF-κB, ATF6, and SREBPs, which operate in a number of physio-pathological molecular mechanisms. Given the close connection of SND1 with the control of cell growth and malignant transformation, SND1 has become a novel candidate for cancer treatment and a valid molecular biomarker for prognosis and diagnosis in some cancers (32, 37, 38, 97). The inhibition of *SND1* gene and SND1 protein is at present the focus of a number of clinical investigations.

A selective inhibitor of the SND1 endonuclease activity, the 3′5′-deoxythymidine bisphosphate, has succeeded in reducing hepatocellular carcinoma cells proliferation and inflammation, as well as *in vivo* tumor formation (36, 37). This inhibitor might serve as a specific nontoxic antitumor agent as it impairs the enzymatic activity-driven functions of SND1 but is ineffective against those that are nuclease-independent. Viable options to investigate the effect of SND1 inhibition in overcoming cancer progression are SND1 targeting by nanoparticles-encapsulated specific siRNAs (98) as well as the generation of full or conditional *SND1* knockout mouse models. These approaches to SND1 deletion would provide an overview about the biological significance of SND1 and its involvement in oncogenic processes and normal development.

Aforementioned findings about the *SND1* promoter regulation may have implications in the development of therapeutic interventions capable of acting on the transcription of *SND1* gene directly. Regulatory elements and transcription factors that transcriptionally upregulate SND1 are commonly involved in oncogenic processes. Their inhibition by genome editing or drugs exerts solid antitumor effects (99, 100). An important question to address is how the inhibition of each transcription factor can affect the oncogenic functions of SND1 and whether their combination with SND1 endonuclease inhibitors may offer clinical benefits.

A particularly promising aspect to challenge as potential targets to downregulate SND1 overexpression in tumor cells is the potential presence of functional G-quadruplex forming motifs in the promoter region of *SND1*. A variety of small ligands have been reported to induce the formation and stabilization of G-quadruplex structures in most oncogenes and might be used as anti-cancer drugs (87, 88). However, whether the G-quadruplex structures in the DNA may have a role, either inhibitory or activatory, in the control of SND1 expression is unknown and a hypothesis that needs to be interrogated.

In conclusion, the functional diversity of *SND1* marks this gene as interesting for further analysis in relation with the multiple levels of regulation of SND1 protein expression. Analysis of *SND1* orthologues using approaches such us gene overexpression, silencing, mutant analysis, transgenic and organ-specific knockout animals, could help to elucidate the role of SND1 in normal cell growth, development and oncogenesis, and to assess whether the distorted SND1 expression level is the cause or the consequence of the oncogenic environment. This does not rule out the possibility that the encoded protein SND1 has protective roles throughout a series of effectors in cells undergoing certain stress types. The reported findings show features supporting both sides of the coin.

## Author Contributions

MJM, YC, and BO conceived the study and wrote the manuscript. All authors approved the final manuscript.

### Conflict of Interest Statement

The authors declare that the research was conducted in the absence of any commercial or financial relationships that could be construed as a potential conflict of interest.

## Abbreviations

ATF: activating transcription factor
ER: endoplasmic reticulum
miRNA: microRNA
NF-κB: necrosis factor κB
SND1: staphylococcal nuclease and tudor domain containing 1
SRE: sterol response element
SREBP: SRE binding protein
TNFα: tumor necrosis factor α
UPR: unfolded protein response
XBP1: X-box binding protein 1.
